# Editorial: The Vasopressin System and Behavior

**DOI:** 10.3389/fendo.2018.00438

**Published:** 2018-08-06

**Authors:** Heather K. Caldwell, Aras Petrulis

**Affiliations:** ^1^Laboratory of Neuroendocrinology and Behavior, Department of Biological Sciences and School of Biomedical Sciences, Kent State University, Kent, OH, United States; ^2^Neuroscience Institute, Georgia State University, Atlanta, GA, United States

**Keywords:** vasopressin, oxytocin, vasotocin, behavior, brain

The awarding of the 1955 Nobel Prize in Chemistry to Vincent du Vigneaud, in part for his isolation and synthesis of the neuropeptide arginine vasopressin (Avp), ushered in a new era of research focused on the ways in which Avp (and its evolutionary precursor vasotocin (Avt)) regulate physiology and behavior (1, 2). While early studies focused primarily on the peripheral effects of these neuropeptides, it soon emerged that Avp, and its homologs, are important neuromodulators of behavior; a PubMed search indicates a steady rise in the number of published papers on this topic each year since the mid-1950s, with over 120 papers published per year since 2012. No doubt this continued enthusiasm is due, in part, to the evolutionarily ancient and highly-conserved nature of Avp-like molecules across vertebrate taxa (3). As a result, there is now a tremendous richness in this literature, both in species breadth and mechanistic depth. It was with this in mind that we organized this research topic on “The Vasopressin System and Behavior,” in which we hoped to bring together a diversity of perspectives outlining areas of consensus and divergence, as well as to facilitate discussion.

Within this collection, numerous reviews and empirical papers explore the complexity of the Avp and Avt systems in the context of different behavioral systems and taxa. From these papers, several broad themes and some “calls to action” have emerged. First, although it is clear that Avp/Avt are key regulators of social and emotional behavior [e.g., (4–7)], there is a lack of consensus regarding the contexts under which they modulate behavior. Thus, this remains an area requiring even deeper scrutiny. For example, Carter reviews how complex interactions between the Avp and oxytocin systems can affect an animal's responses within differing emotional and social contexts. Second, a broader look at how Avp/Avt influences brain systems, rather than just individual brain regions, is required for further progress. This is underscored by papers from Ophir and Phelps et al. that outline the potential for Avp and oxytocin to alter cortical and hippocampal dynamics underlying complex social space use. Third, even though sex-differences in response to Avp have been frequently noted in the literature [e.g., (8–11)], there are still behavioral domains and taxa in which more critical evaluation of sex differences in the Avp/Avt systems are needed Taylor et al.; Wilczynski et al.; Tickerhoof and Smith; Terranova et al. Fourth, as Simmons et al., Murgatroyd et al., and Baran demonstrate, there is an important role for Avp/Avt throughout behavioral development that is currently under-examined. Fifth, as is explored in Caldwell et al. and Terranova et al., continued consideration of receptor dynamics and subtypes that mediate Avp effects will be needed to more fully understand the relationship between Avp release and its locus of action. Sixth, given the ancient origin of Avp/Avt, a continued commitment to explore their roles across species will aid in finding areas of convergence and divergence in behavioral function. This diversity of Avp/Avt behavioral action across different species is nicely illustrated by contributions from Baran, Rodriguez-Santiago et al., Wilczynski et al., and Latzman et al. Lastly, the Avp system is clearly important in regulating human and non-human primate behavior, as described by Latzman et al., Murgatroyd et al., Patisaul, Rilling et al., Price et al., Taylor et al. However, compared to the avalanche of data on oxytocin effects in humans, much less is known and understood regarding the how, the where, and the when of Avp effects on primate social behavior. Thus, there is a need for further detailed examinations of Avp's influence on behavior and cognition, especially within the context of human health.

We are hopeful that these diverse and thoughtful papers will be utilized by both new and more seasoned investigators to guide their work and will spark new discussions about the role of the Avp/Avt systems in behavioral regulation. Given the complexity of these systems, the diversity of species studied, and the numerous behaviors they regulate, it appears that even 60 years after the structural definition of Avp, much work still remains to be done.

## Author contributions

All authors listed have made a substantial, direct and intellectual contribution to the work, and approved it for publication.

### Conflict of interest statement

The authors declare that the research was conducted in the absence of any commercial or financial relationships that could be construed as a potential conflict of interest.
